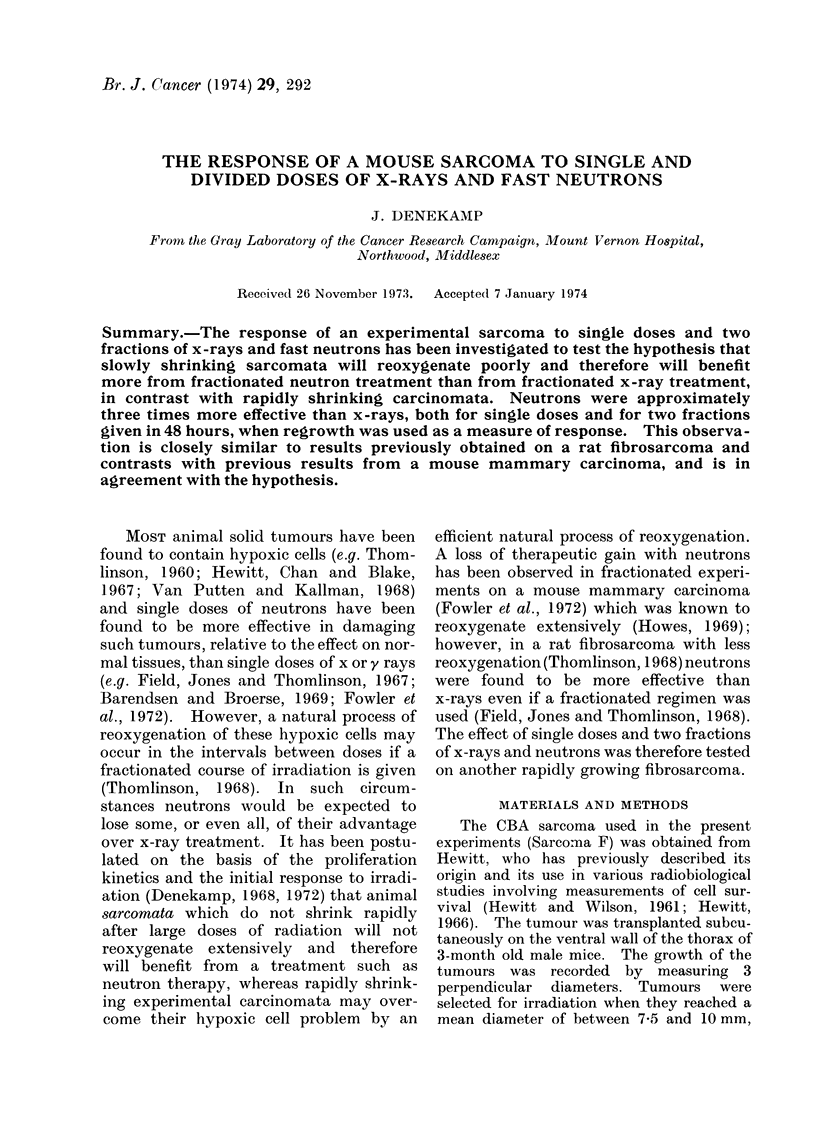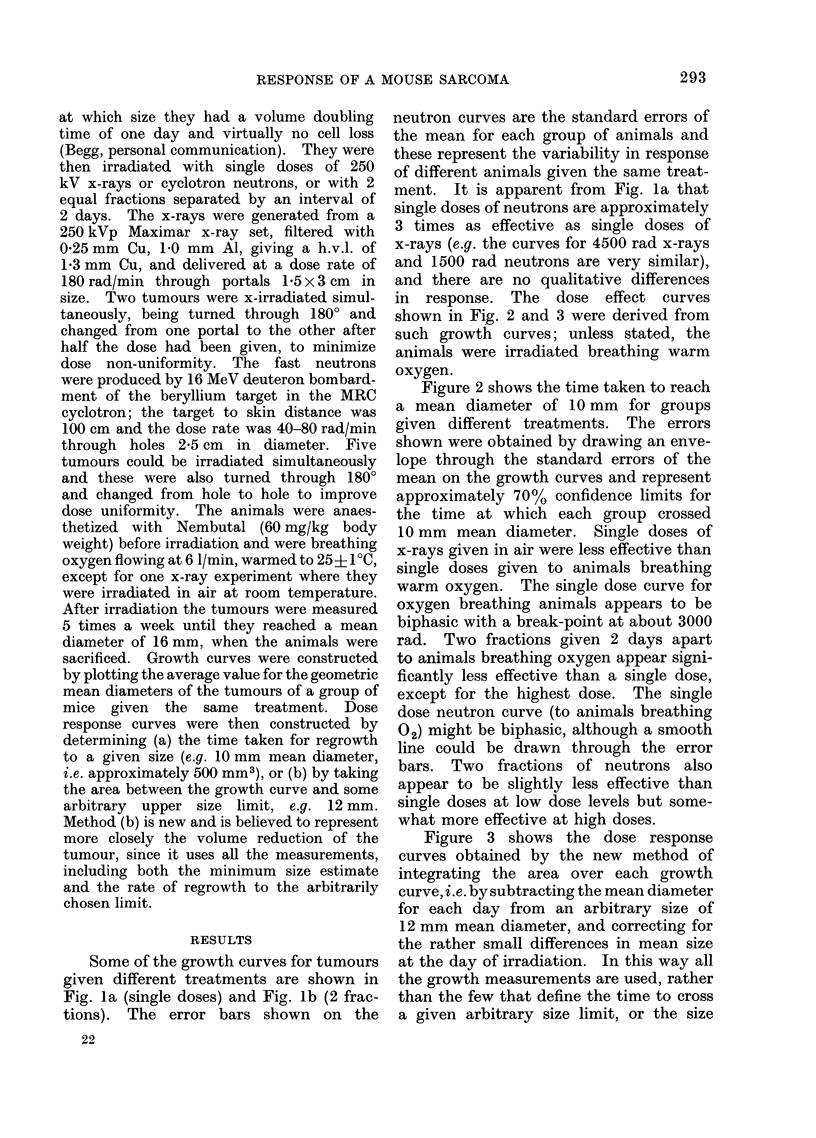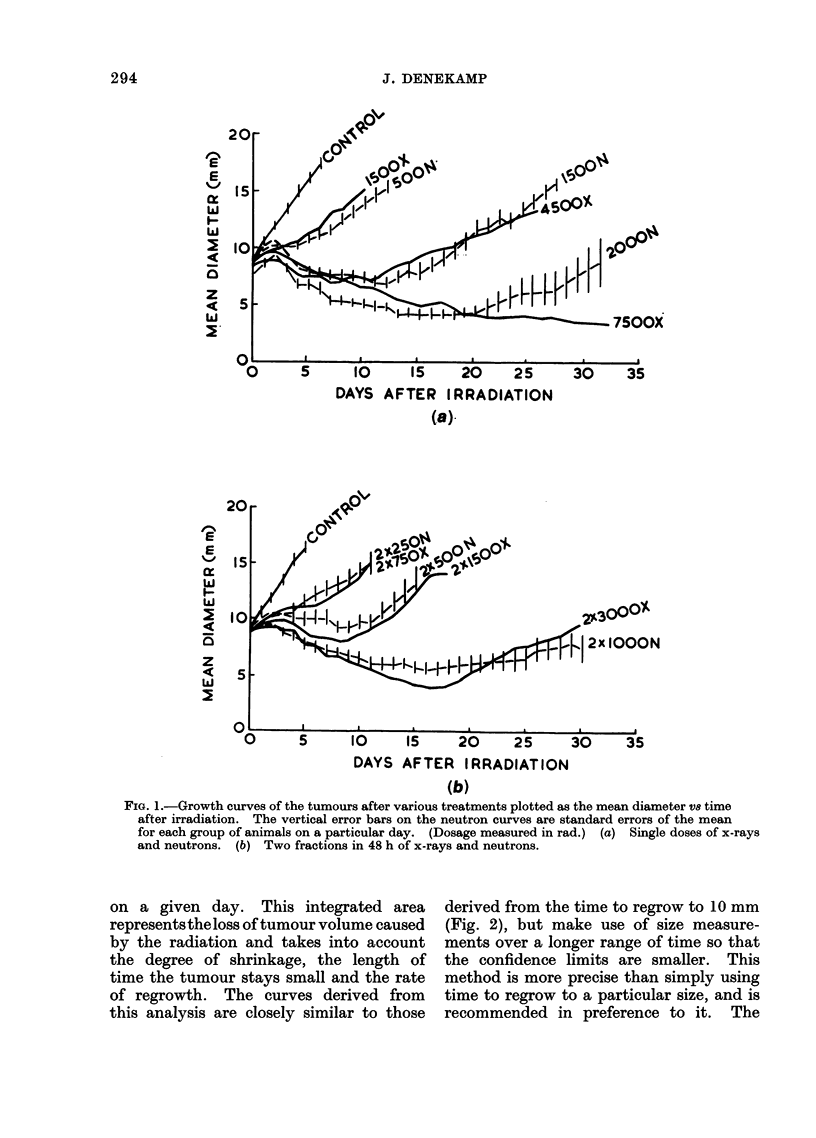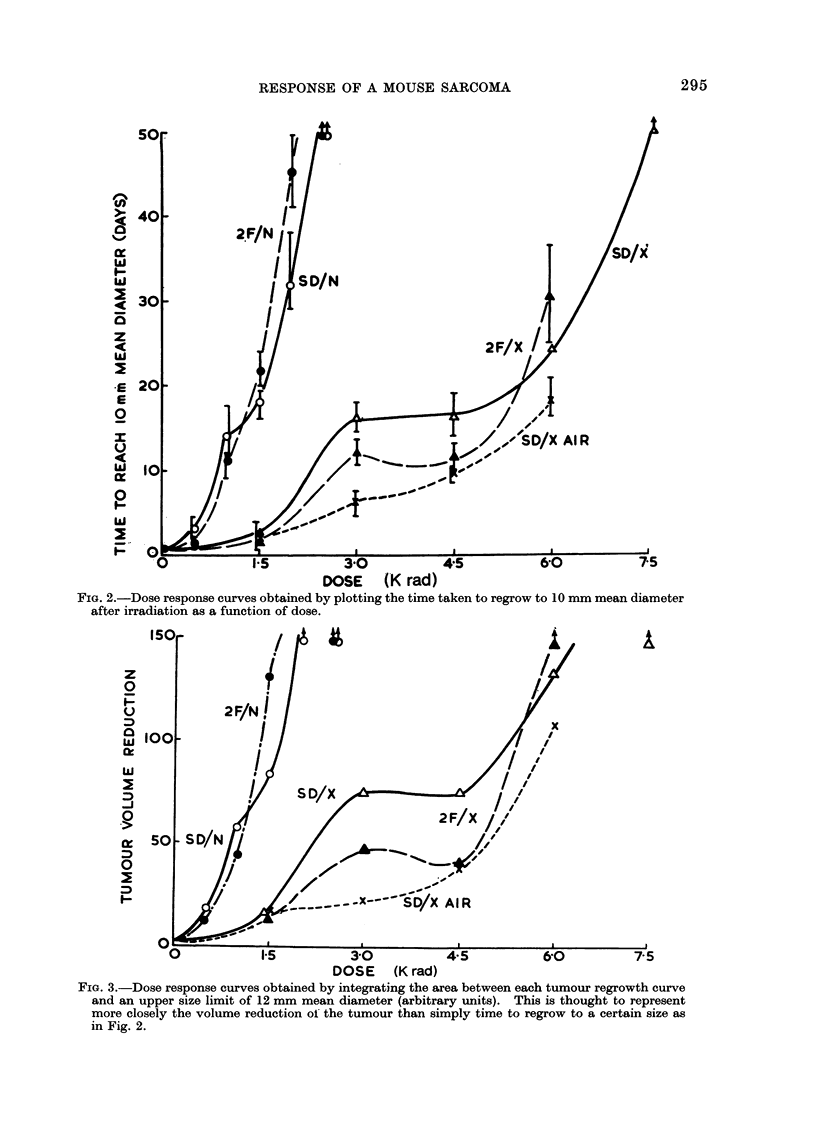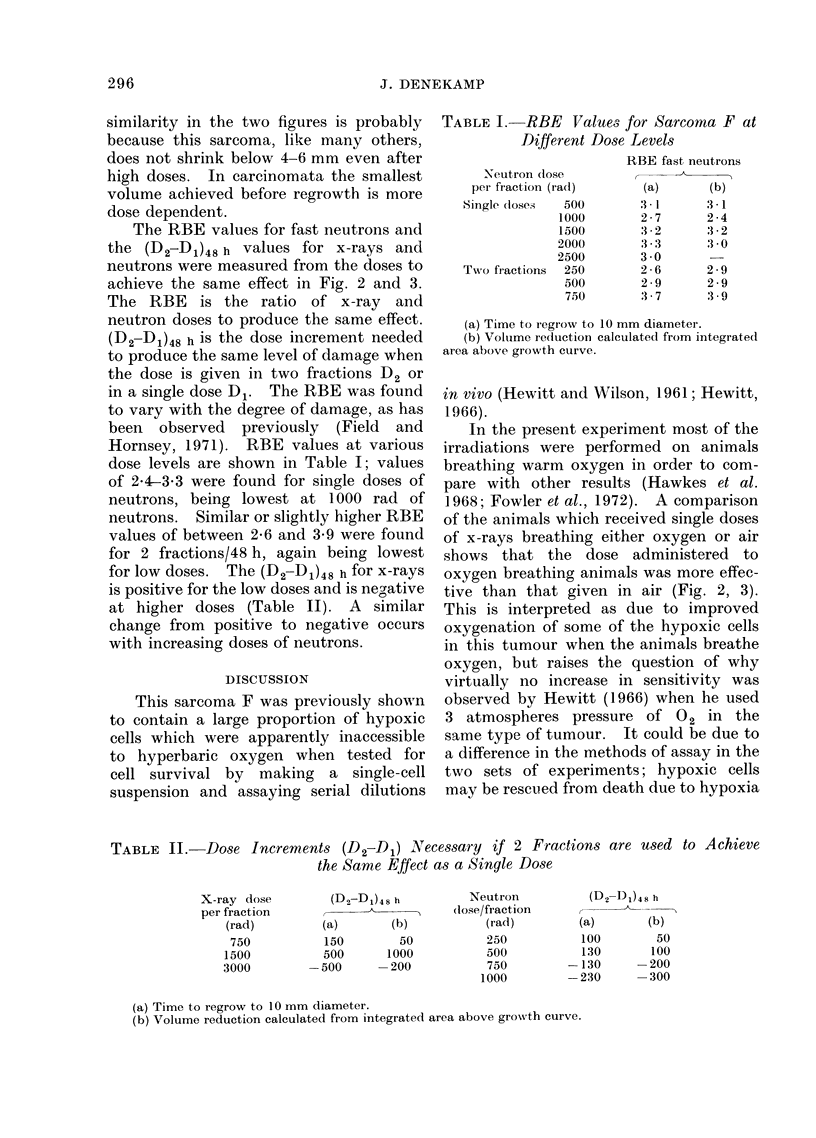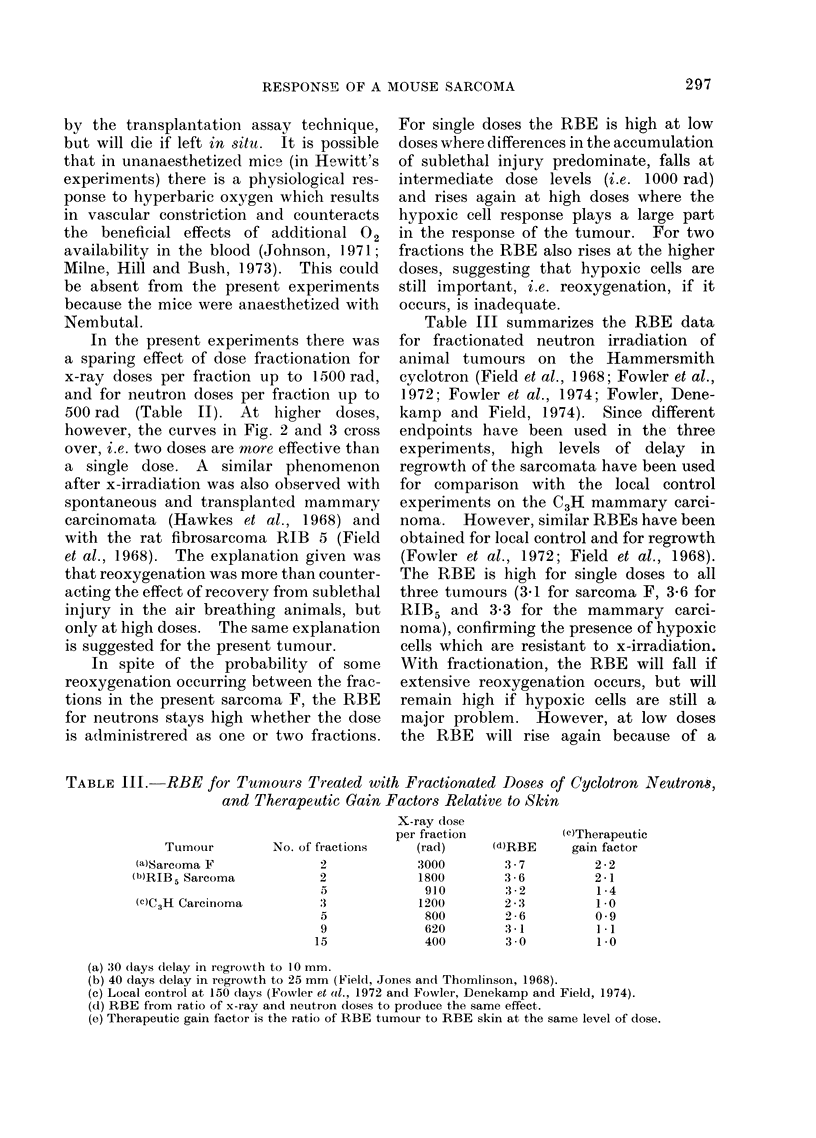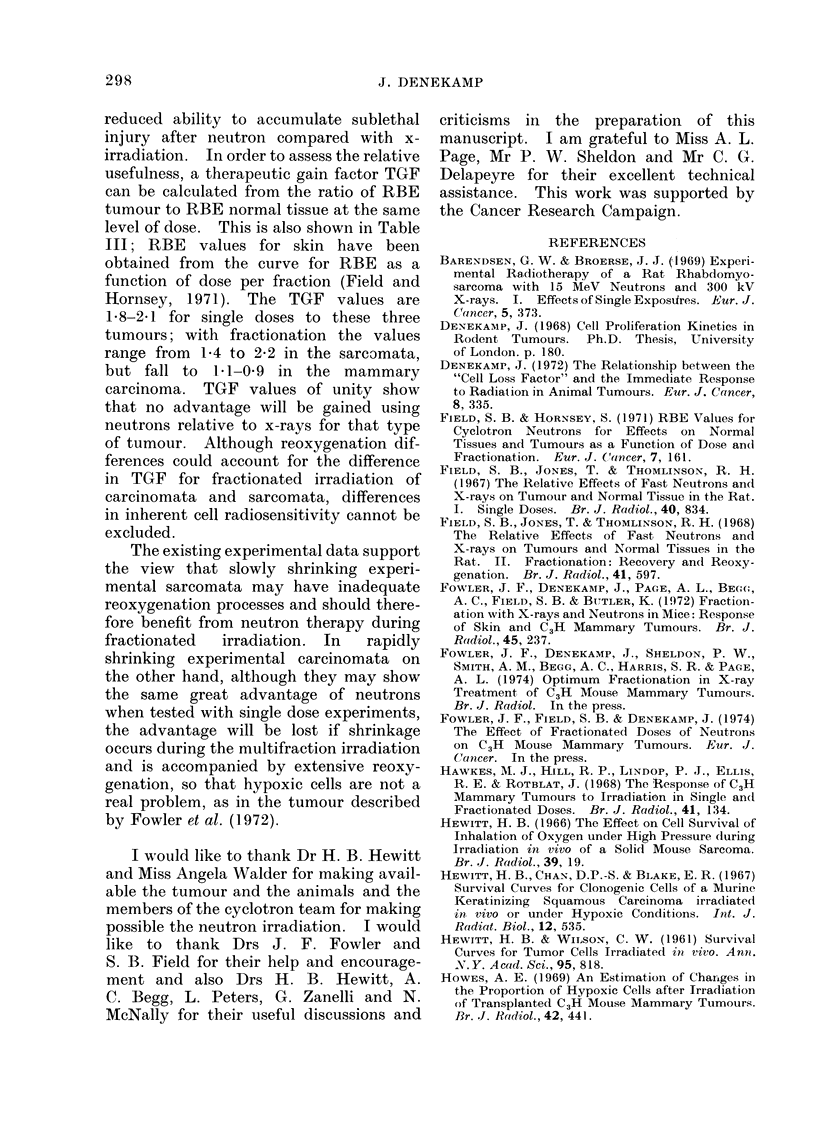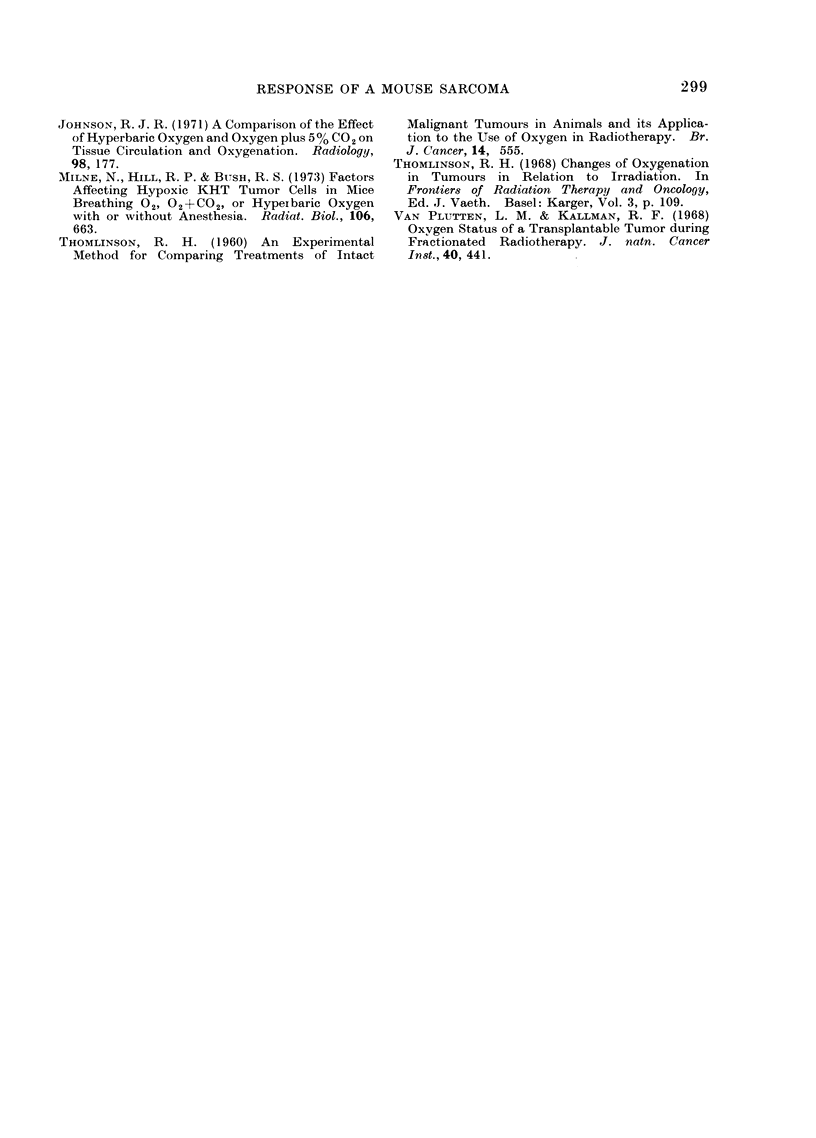# The Response of a Mouse Sarcoma to Single and Divided Doses of X-rays and Fast Neutrons

**DOI:** 10.1038/bjc.1974.69

**Published:** 1974-04

**Authors:** J. Denekamp

## Abstract

The response of an experimental sarcoma to single doses and two fractions of x-rays and fast neutrons has been investigated to test the hypothesis that slowly shrinking sarcomata will reoxygenate poorly and therefore will benefit more from fractionated neutron treatment than from fractionated x-ray treatment, in contrast with rapidly shrinking carcinomata. Neutrons were approximately three times more effective than x-rays, both for single doses and for two fractions given in 48 hours, when regrowth was used as a measure of response. This observation is closely similar to results previously obtained on a rat fibrosarcoma and contrasts with previous results from a mouse mammary carcinoma, and is in agreement with the hypothesis.


*Br. J. Cancer* (1974) **29**, 292

### THE RESPONSE OF A MOUSE SARCOMA TO SINGLE AND DIVIDED DOSES OF X-RAYS AND FAST NEUTRONS

J. DENEKAMP

*From the Gray Laboratory of the Cancer Research Campaign, Mount Vernon Hospital, Northwood, Middlesex*

Received 26 November 1973. Accepted 7 January 1974

**Summary.—The response of an experimental sarcoma to single doses and two fractions of x-rays and fast neutrons has been investigated to test the hypothesis that slowly shrinking sarcomata will reoxygenate poorly and therefore will benefit more from fractionated neutron treatment than from fractionated x-ray treatment, in contrast with rapidly shrinking carcinomata. Neutrons were approximately three times more effective than x-rays, both for single doses and for two fractions given in 48 hours, when regrowth was used as a measure of response. This observation is closely similar to results previously obtained on a rat fibrosarcoma and contrasts with previous results from a mouse mammary carcinoma, and is in agreement with the hypothesis.**

MOST animal solid tumours have been found to contain hypoxic cells (*e.g.* Thomlinson, 1960; Hewitt, Chan and Blake, 1967; Van Putten and Kallman, 1968) and single doses of neutrons have been found to be more effective in damaging such tumours, relative to the effect on normal tissues, than single doses of x or $\gamma$ rays (*e.g.* Field, Jones and Thomlinson, 1967; Barendsen and Broerse, 1969; Fowler *et al.*, 1972). However, a natural process of reoxygenation of these hypoxic cells may occur in the intervals between doses if a fractionated course of irradiation is given (Thomlinson, 1968). In such circumstances neutrons would be expected to lose some, or even all, of their advantage over x-ray treatment. It has been postulated on the basis of the proliferation kinetics and the initial response to irradiation (Denekamp, 1968, 1972) that animal *sarcomata* which do not shrink rapidly after large doses of radiation will not reoxygenate extensively and therefore will benefit from a treatment such as neutron therapy, whereas rapidly shrinking experimental carcinomata may overcome their hypoxic cell problem by an efficient natural process of reoxygenation. A loss of therapeutic gain with neutrons has been observed in fractionated experiments on a mouse mammary carcinoma (Fowler *et al.*, 1972) which was known to reoxygenate extensively (Howes, 1969); however, in a rat fibrosarcoma with less reoxygenation (Thomlinson, 1968) neutrons were found to be more effective than x-rays even if a fractionated regimen was used (Field, Jones and Thomlinson, 1968). The effect of single doses and two fractions of x-rays and neutrons was therefore tested on another rapidly growing fibrosarcoma.

### MATERIALS AND METHODS

The CBA sarcoma used in the present experiments (Sarcoma F) was obtained from Hewitt, who has previously described its origin and its use in various radiobiological studies involving measurements of cell survival (Hewitt and Wilson, 1961; Hewitt, 1966). The tumour was transplanted subcutaneously on the ventral wall of the thorax of 3-month old male mice. The growth of the tumours was recorded by measuring 3 perpendicular diameters. Tumours were selected for irradiation when they reached a mean diameter of between 7·5 and 10 mm, at which size they had a volume doubling time of one day and virtually no cell loss (Begg, personal communication). They were then irradiated with single doses of 250 kV x-rays or cyclotron neutrons, or with 2 equal fractions separated by an interval of 2 days. The x-rays were generated from a 250 kVp Maximar x-ray set, filtered with 0·25 mm Cu, 1·0 mm Al, giving a h.v.l. of 1·3 mm Cu, and delivered at a dose rate of 180 rad/min through portals $1{\cdot}5 \times 3$ cm in size. Two tumours were x-irradiated simultaneously, being turned through 180° and changed from one portal to the other after half the dose had been given, to minimize dose non-uniformity. The fast neutrons were produced by 16 MeV deuteron bombardment of the beryllium target in the MRC cyclotron; the target to skin distance was 100 cm and the dose rate was 40–80 rad/min through holes 2·5 cm in diameter. Five tumours could be irradiated simultaneously and these were also turned through 180° and changed from hole to hole to improve dose uniformity. The animals were anaesthetized with Nembutal (60 mg/kg body weight) before irradiation and were breathing oxygen flowing at 6 l/min, warmed to $25 \pm 1$ °C, except for one x-ray experiment where they were irradiated in air at room temperature. After irradiation the tumours were measured 5 times a week until they reached a mean diameter of 16 mm, when the animals were sacrificed. Growth curves were constructed by plotting the average value for the geometric mean diameters of the tumours of a group of mice given the same treatment. Dose response curves were then constructed by determining (a) the time taken for regrowth to a given size (*e.g.* 10 mm mean diameter, *i.e.* approximately 500 $mm^3$), or (b) by taking the area between the growth curve and some arbitrary upper size limit, *e.g.* 12 mm. Method (b) is new and is believed to represent more closely the volume reduction of the tumour, since it uses all the measurements, including both the minimum size estimate and the rate of regrowth to the arbitrarily chosen limit.

### RESULTS

Some of the growth curves for tumours given different treatments are shown in Fig. 1a (single doses) and Fig. 1b (2 fractions). The error bars shown on the neutron curves are the standard errors of the mean for each group of animals and these represent the variability in response of different animals given the same treatment. It is apparent from Fig. 1a that single doses of neutrons are approximately 3 times as effective as single doses of x-rays (*e.g.* the curves for 4500 rad x-rays and 1500 rad neutrons are very similar), and there are no qualitative differences in response. The dose effect curves shown in Fig. 2 and 3 were derived from such growth curves; unless stated, the animals were irradiated breathing warm oxygen.

Figure 2 shows the time taken to reach a mean diameter of 10 mm for groups given different treatments. The errors shown were obtained by drawing an envelope through the standard errors of the mean on the growth curves and represent approximately 70% confidence limits for the time at which each group crossed 10 mm mean diameter. Single doses of x-rays given in air were less effective than single doses given to animals breathing warm oxygen. The single dose curve for oxygen breathing animals appears to be biphasic with a break-point at about 3000 rad. Two fractions given 2 days apart to animals breathing oxygen appear significantly less effective than a single dose, except for the highest dose. The single dose neutron curve (to animals breathing $O_2$) might be biphasic, although a smooth line could be drawn through the error bars. Two fractions of neutrons also appear to be slightly less effective than single doses at low dose levels but somewhat more effective at high doses.

Figure 3 shows the dose response curves obtained by the new method of integrating the area over each growth curve, *i.e.* by subtracting the mean diameter for each day from an arbitrary size of 12 mm mean diameter, and correcting for the rather small differences in mean size at the day of irradiation. In this way all the growth measurements are used, rather than the few that define the time to cross a given arbitrary size limit, or the size

(a)

(b)

FIG. 1.—Growth curves of the tumours after various treatments plotted as the mean diameter *vs* time after irradiation. The vertical error bars on the neutron curves are standard errors of the mean for each group of animals on a particular day. (Dosage measured in rad.) (*a*) Single doses of x-rays and neutrons. (*b*) Two fractions in 48 h of x-rays and neutrons.

on a given day. This integrated area represents the loss of tumour volume caused by the radiation and takes into account the degree of shrinkage, the length of time the tumour stays small and the rate of regrowth. The curves derived from this analysis are closely similar to those derived from the time to regrow to 10 mm (Fig. 2), but make use of size measurements over a longer range of time so that the confidence limits are smaller. This method is more precise than simply using time to regrow to a particular size, and is recommended in preference to it. The

FIG. 2.—Dose response curves obtained by plotting the time taken to regrow to 10 mm mean diameter after irradiation as a function of dose.

FIG. 3.—Dose response curves obtained by integrating the area between each tumour regrowth curve and an upper size limit of 12 mm mean diameter (arbitrary units). This is thought to represent more closely the volume reduction of the tumour than simply time to regrow to a certain size as in Fig. 2.

similarity in the two figures is probably because this sarcoma, like many others, does not shrink below 4–6 mm even after high doses. In carcinomata the smallest volume achieved before regrowth is more dose dependent.

The RBE values for fast neutrons and the $(D_2–D_1)_{48\ h}$ values for x-rays and neutrons were measured from the doses to achieve the same effect in Fig. 2 and 3. The RBE is the ratio of x-ray and neutron doses to produce the same effect. $(D_2–D_1)_{48\ h}$ is the dose increment needed to produce the same level of damage when the dose is given in two fractions $D_2$ or in a single dose $D_1$. The RBE was found to vary with the degree of damage, as has been observed previously (Field and Hornsey, 1971). RBE values at various dose levels are shown in Table I; values of 2·4–3·3 were found for single doses of neutrons, being lowest at 1000 rad of neutrons. Similar or slightly higher RBE values of between 2·6 and 3·9 were found for 2 fractions/48 h, again being lowest for low doses. The $(D_2–D_1)_{48\ h}$ for x-rays is positive for the low doses and is negative at higher doses (Table II). A similar change from positive to negative occurs with increasing doses of neutrons.

TABLE I.—*RBE Values for Sarcoma F at Different Dose Levels*

| Neutron dose per fraction (rad) | | RBE fast neutrons (a) | (b) |
|---|---|---|---|
| Single doses | 500 | 3·1 | 3·1 |
| | 1000 | 2·7 | 2·4 |
| | 1500 | 3·2 | 3·2 |
| | 2000 | 3·3 | 3·0 |
| | 2500 | 3·0 | — |
| Two fractions | 250 | 2·6 | 2·9 |
| | 500 | 2·9 | 2·9 |
| | 750 | 3·7 | 3·9 |

(a) Time to regrow to 10 mm diameter.
(b) Volume reduction calculated from integrated area above growth curve.

### DISCUSSION

This sarcoma F was previously shown to contain a large proportion of hypoxic cells which were apparently inaccessible to hyperbaric oxygen when tested for cell survival by making a single-cell suspension and assaying serial dilutions *in vivo* (Hewitt and Wilson, 1961; Hewitt, 1966).

In the present experiment most of the irradiations were performed on animals breathing warm oxygen in order to compare with other results (Hawkes *et al.* 1968; Fowler *et al.*, 1972). A comparison of the animals which received single doses of x-rays breathing either oxygen or air shows that the dose administered to oxygen breathing animals was more effective than that given in air (Fig. 2, 3). This is interpreted as due to improved oxygenation of some of the hypoxic cells in this tumour when the animals breathe oxygen, but raises the question of why virtually no increase in sensitivity was observed by Hewitt (1966) when he used 3 atmospheres pressure of $O_2$ in the same type of tumour. It could be due to a difference in the methods of assay in the two sets of experiments; hypoxic cells may be rescued from death due to hypoxia

TABLE II.—*Dose Increments* $(D_2–D_1)$ *Necessary if 2 Fractions are used to Achieve the Same Effect as a Single Dose*

| X-ray dose per fraction (rad) | $(D_2–D_1)_{48\ h}$ (a) | (b) | Neutron dose/fraction (rad) | $(D_2–D_1)_{48\ h}$ (a) | (b) |
|---|---|---|---|---|---|
| 750 | 150 | 50 | 250 | 100 | 50 |
| 1500 | 500 | 1000 | 500 | 130 | 100 |
| 3000 | −500 | −200 | 750 | −130 | −200 |
| | | | 1000 | −230 | −300 |

(a) Time to regrow to 10 mm diameter.
(b) Volume reduction calculated from integrated area above growth curve.

by the transplantation assay technique, but will die if left *in situ*. It is possible that in unanaesthetized mice (in Hewitt's experiments) there is a physiological response to hyperbaric oxygen which results in vascular constriction and counteracts the beneficial effects of additional $O_2$ availability in the blood (Johnson, 1971; Milne, Hill and Bush, 1973). This could be absent from the present experiments because the mice were anaesthetized with Nembutal.

In the present experiments there was a sparing effect of dose fractionation for x-ray doses per fraction up to 1500 rad, and for neutron doses per fraction up to 500 rad (Table II). At higher doses, however, the curves in Fig. 2 and 3 cross over, *i.e.* two doses are *more* effective than a single dose. A similar phenomenon after x-irradiation was also observed with spontaneous and transplanted mammary carcinomata (Hawkes *et al.*, 1968) and with the rat fibrosarcoma RIB 5 (Field *et al.*, 1968). The explanation given was that reoxygenation was more than counteracting the effect of recovery from sublethal injury in the air breathing animals, but only at high doses. The same explanation is suggested for the present tumour.

In spite of the probability of some reoxygenation occurring between the fractions in the present sarcoma F, the RBE for neutrons stays high whether the dose is administrered as one or two fractions. For single doses the RBE is high at low doses where differences in the accumulation of sublethal injury predominate, falls at intermediate dose levels (*i.e.* 1000 rad) and rises again at high doses where the hypoxic cell response plays a large part in the response of the tumour. For two fractions the RBE also rises at the higher doses, suggesting that hypoxic cells are still important, *i.e.* reoxygenation, if it occurs, is inadequate.

Table III summarizes the RBE data for fractionated neutron irradiation of animal tumours on the Hammersmith cyclotron (Field *et al.*, 1968; Fowler *et al.*, 1972; Fowler *et al.*, 1974; Fowler, Denekamp and Field, 1974). Since different endpoints have been used in the three experiments, high levels of delay in regrowth of the sarcomata have been used for comparison with the local control experiments on the $C_3H$ mammary carcinoma. However, similar RBEs have been obtained for local control and for regrowth (Fowler *et al.*, 1972; Field *et al.*, 1968). The RBE is high for single doses to all three tumours (3·1 for sarcoma F, 3·6 for $RIB_5$ and 3·3 for the mammary carcinoma), confirming the presence of hypoxic cells which are resistant to x-irradiation. With fractionation, the RBE will fall if extensive reoxygenation occurs, but will remain high if hypoxic cells are still a major problem. However, at low doses the RBE will rise again because of a

TABLE III.—*RBE for Tumours Treated with Fractionated Doses of Cyclotron Neutrons, and Therapeutic Gain Factors Relative to Skin*

| Tumour | No. of fractions | X-ray dose per fraction (rad) | [d]RBE | [e]Therapeutic gain factor |
|---|---|---|---|---|
| [a]Sarcoma F | 2 | 3000 | 3·7 | 2·2 |
| [b]$RIB_5$ Sarcoma | 2 | 1800 | 3·6 | 2·1 |
| | 5 | 910 | 3·2 | 1·4 |
| [c]$C_3H$ Carcinoma | 3 | 1200 | 2·3 | 1·0 |
| | 5 | 800 | 2·6 | 0·9 |
| | 9 | 620 | 3·1 | 1·1 |
| | 15 | 400 | 3·0 | 1·0 |

(a) 30 days delay in regrowth to 10 mm.
(b) 40 days delay in regrowth to 25 mm (Field, Jones and Thomlinson, 1968).
(c) Local control at 150 days (Fowler *et al.*, 1972 and Fowler, Denekamp and Field, 1974).
(d) RBE from ratio of x-ray and neutron doses to produce the same effect.
(e) Therapeutic gain factor is the ratio of RBE tumour to RBE skin at the same level of dose.

reduced ability to accumulate sublethal injury after neutron compared with x-irradiation. In order to assess the relative usefulness, a therapeutic gain factor TGF can be calculated from the ratio of RBE tumour to RBE normal tissue at the same level of dose. This is also shown in Table III; RBE values for skin have been obtained from the curve for RBE as a function of dose per fraction (Field and Hornsey, 1971). The TGF values are 1·8–2·1 for single doses to these three tumours; with fractionation the values range from 1·4 to 2·2 in the sarcomata, but fall to 1·1–0·9 in the mammary carcinoma. TGF values of unity show that no advantage will be gained using neutrons relative to x-rays for that type of tumour. Although reoxygenation differences could account for the difference in TGF for fractionated irradiation of carcinomata and sarcomata, differences in inherent cell radiosensitivity cannot be excluded.

The existing experimental data support the view that slowly shrinking experimental sarcomata may have inadequate reoxygenation processes and should therefore benefit from neutron therapy during fractionated irradiation. In rapidly shrinking experimental carcinomata on the other hand, although they may show the same great advantage of neutrons when tested with single dose experiments, the advantage will be lost if shrinkage occurs during the multifraction irradiation and is accompanied by extensive reoxygenation, so that hypoxic cells are not a real problem, as in the tumour described by Fowler *et al.* (1972).

I would like to thank Dr H. B. Hewitt and Miss Angela Walder for making available the tumour and the animals and the members of the cyclotron team for making possible the neutron irradiation. I would like to thank Drs J. F. Fowler and S. B. Field for their help and encouragement and also Drs H. B. Hewitt, A. C. Begg, L. Peters, G. Zanelli and N. McNally for their useful discussions and criticisms in the preparation of this manuscript. I am grateful to Miss A. L. Page, Mr P. W. Sheldon and Mr C. G. Delapeyre for their excellent technical assistance. This work was supported by the Cancer Research Campaign.

### REFERENCES

Barendsen, G. W. & Broerse, J. J. (1969) Experimental Radiotherapy of a Rat Rhabdomyosarcoma with 15 MeV Neutrons and 300 kV X-rays. I. Effects of Single Exposures. *Eur. J. Cancer*, **5**, 373.

Denekamp, J. (1968) Cell Proliferation Kinetics in Rodent Tumours. Ph.D. Thesis, University of London. p. 180.

Denekamp, J. (1972) The Relationship between the "Cell Loss Factor" and the Immediate Response to Radiation in Animal Tumours. *Eur. J. Cancer*, **8**, 335.

Field, S. B. & Hornsey, S. (1971) RBE Values for Cyclotron Neutrons for Effects on Normal Tissues and Tumours as a Function of Dose and Fractionation. *Eur. J. Cancer*, **7**, 161.

Field, S. B., Jones, T. & Thomlinson, R. H. (1967) The Relative Effects of Fast Neutrons and X-rays on Tumour and Normal Tissue in the Rat. I. Single Doses. *Br. J. Radiol.*, **40**, 834.

Field, S. B., Jones, T. & Thomlinson, R. H. (1968) The Relative Effects of Fast Neutrons and X-rays on Tumours and Normal Tissues in the Rat. II. Fractionation: Recovery and Reoxygenation. *Br. J. Radiol.*, **41**, 597.

Fowler, J. F., Denekamp, J., Page, A. L., Begg, A. C., Field, S. B. & Butler, K. (1972) Fractionation with X-rays and Neutrons in Mice: Response of Skin and $C_3H$ Mammary Tumours. *Br. J. Radiol.*, **45**, 237.

Fowler, J. F., Denekamp, J., Sheldon, P. W., Smith, A. M., Begg, A. C., Harris, S. R. & Page, A. L. (1974) Optimum Fractionation in X-ray Treatment of $C_3H$ Mouse Mammary Tumours. *Br. J. Radiol.* In the press.

Fowler, J. F., Field, S. B. & Denekamp, J. (1974) The Effect of Fractionated Doses of Neutrons on $C_3H$ Mouse Mammary Tumours. *Eur. J. Cancer*. In the press.

Hawkes, M. J., Hill, R. P., Lindop, P. J., Ellis, R. E. & Rotblat, J. (1968) The Response of $C_3H$ Mammary Tumours to Irradiation in Single and Fractionated Doses. *Br. J. Radiol.*, **41**, 134.

Hewitt, H. B. (1966) The Effect on Cell Survival of Inhalation of Oxygen under High Pressure during Irradiation *in vivo* of a Solid Mouse Sarcoma. *Br. J. Radiol.*, **39**, 19.

Hewitt, H. B., Chan, D.P.-S. & Blake, E. R. (1967) Survival Curves for Clonogenic Cells of a Murine Keratinizing Squamous Carcinoma irradiated *in vivo* or under Hypoxic Conditions. *Int. J. Radiat. Biol.*, **12**, 535.

Hewitt, H. B. & Wilson, C. W. (1961) Survival Curves for Tumor Cells Irradiated *in vivo*. *Ann. N.Y. Acad. Sci.*, **95**, 818.

Howes, A. E. (1969) An Estimation of Changes in the Proportion of Hypoxic Cells after Irradiation of Transplanted $C_3H$ Mouse Mammary Tumours. *Br. J. Radiol.*, **42**, 441.

JOHNSON, R. J. R. (1971) A Comparison of the Effect of Hyperbaric Oxygen and Oxygen plus 5% $CO_2$ on Tissue Circulation and Oxygenation. *Radiology*, **98**, 177.

MILNE, N., HILL, R. P. & BUSH, R. S. (1973) Factors Affecting Hypoxic KHT Tumor Cells in Mice Breathing $O_2$, $O_2+CO_2$, or Hyperbaric Oxygen with or without Anesthesia. *Radiat. Biol.*, **106**, 663.

THOMLINSON, R. H. (1960) An Experimental Method for Comparing Treatments of Intact Malignant Tumours in Animals and its Application to the Use of Oxygen in Radiotherapy. *Br. J. Cancer*, **14**, 555.

THOMLINSON, R. H. (1968) Changes of Oxygenation in Tumours in Relation to Irradiation. In *Frontiers of Radiation Therapy and Oncology*, Ed. J. Vaeth. Basel: Karger, Vol. 3, p. 109.

VAN PLUTTEN, L. M. & KALLMAN, R. F. (1968) Oxygen Status of a Transplantable Tumor during Fractionated Radiotherapy. *J. natn. Cancer Inst.*, **40**, 441.